# Validation of In-Shoe Force Sensors during Loaded Walking in Military Personnel

**DOI:** 10.3390/s23146465

**Published:** 2023-07-17

**Authors:** Pui Wah Kong, Muhammad Nur Shahril Iskandar, Ang Hong Koh, Mei Yee Mavis Ho, Cheryl Xue Er Lim

**Affiliations:** 1 Physical Education and Sports Science Academic Group, National Institute of Education, Nanyang Technological University, Singapore 637616, Singapore; s200011@e.ntu.edu.sg (M.N.S.I.); abaddylah@yahoo.com.sg (A.H.K.); mho008@e.ntu.edu.sg (M.Y.M.H.); 2 Centre of Excellence for Soldier Performance, Singapore Armed Forces, Singapore 637901, Singapore; lim_xue_er_cheryl@defence.gov.sg

**Keywords:** loadsol^®^, gait, ground reaction force, load carriage, inclined, declined

## Abstract

The loadsol^®^ wireless in-shoe force sensors can be useful for in-field measurements. However, its accuracy is unknown in the military context, whereby soldiers have to carry heavy loads and walk in military boots. The purpose of this study was to establish the validity of the loadsol^®^ sensors in military personnel during loaded walking on flat, inclined and declined surfaces. Full-time Singapore Armed Forces (SAF) personnel (*n* = 8) walked on an instrumented treadmill on flat, 10° inclined, and 10° declined gradients while carrying heavy loads (25 kg and 35 kg). Normal ground reaction forces (GRF), perpendicular to the contact surface, were simultaneously measured using both the loadsol^®^ sensors inserted in the military boots and the Bertec instrumented treadmill as the gold standard. A total of eight variables of interest were compared between loadsol^®^ and treadmill, including four kinetic (impact peak force, active peak force, impulse, loading rate) and four spatiotemporal (stance time, stride time, cadence, step length) variables. Validity was assessed using Bland–Altman plots and 95% Limits of Agreement (LoA). Bias was calculated as the mean difference between the values obtained from loadsol^®^ and the instrumented treadmill. Results showed similar force-time profiles between loadsol^®^ sensors and the instrumented treadmill. The bias of most variables was generally low, with a narrow range of LoA. The high accuracy and good agreement with standard laboratory equipment suggest that the loadsol^®^ system is a valid tool for measuring normal GRF during walking in military boots under heavy load carriage.

## 1. Introduction

In military operations, soldiers are often required to carry heavy loads and march for a prolonged period of time [1,2]. Given the physically demanding nature of the job, lower limb injuries are common among military populations [3,4]. Measuring the ground reaction forces (GRF) during dynamic weight-bearing activities is useful for quantifying the physical loading placed on the human body. In the military context, for instance, previous studies have used GRF measures to evaluate exoskeletons [5,6], combat boots [7,8], and ankle sprain mechanisms [9].

The gold standard of GRF measurement requires participants to perform movements over force platforms embedded in the ground, typically in a laboratory setting. For example, Bini et al. [10] compared the GRF during overground walking in army recruits when wearing combat boots, sports shoes designed for military training, and regular running shoes. Participants had to walk repeatedly along a 4.3 m walkway to obtain 10 trials per footwear condition with walking speeds monitored by two technicians using time watches. Walking trials had to be repeated if the participants’ feet did not step in full on the force platforms or the walking speed varied beyond the prescribed range. This traditional approach to GRF measurement is time-consuming, requires expensive equipment, and often limits testing to laboratory environments. Recent advancement in wearable technology has opened new opportunities for biomechanical and physiological evaluation, including applications in combat boots [7,11]. Different types of technology have shown promising results in the military context, for example, smart clothing for health and safety monitoring [12], wearables for 24 h heart rate variability monitoring [13], in-shoe plantar pressure measurement systems for gait analysis [11], and instrumented socks to provide real-time audio biofeedback for gait re-training in injured military service members [14]. One notable technology is the loadsol^®^ (novel GmbH, Munich, Germany) wireless in-shoe force sensor for GRF measurements. This system collects normal GRF that acts perpendicular to the contact surface at a sampling rate of 100 Hz. Data are recorded via a mobile phone application without the need for any cables or data loggers, offering a simple and convenient way to perform in-field measurements during military operations.

The accuracy and precision of any wearable sensors should first be considered before adopting them for applications. Previous validation studies on the loadsol^®^ in-shoe force sensors targeted sports-related activities such as running [15,16], hopping [15,17], and stop-jump [17]. Specific to walking, Burns et al. [15] compared loadsol^®^ with an instrumented treadmill when participants walked at 1.39 m/s (5 km/h) and reported good to excellent agreements across all GRF measures. Renner and colleagues [18] also examined the validity of loadsol^®^ against an instrumented treadmill. They concluded that loadsol^®^ is a valid and reliable tool for walking at 1.30 m/s on flat, inclined and declined surfaces. It should be noted that all previous validation studies were conducted without the participants carrying any additional load or equipment [15,16,17,18]. In addition, the loadsol^®^ sensors were validated when participants wore their own shoes [15], running shoes [17,18], or tight-fitted socks without shoes [16]. To the authors’ best knowledge, no studies have validated the accuracy of the loadsol^®^ sensors in the military context, whereby soldiers have to carry heavy loads and walk in military boots. There exist many types of military load carriage systems that are designed for specific purposes [19]. Regardless of the body size of the individuals, soldiers often carry standard-weight military equipment such as backpacks, Kevlar helmets and rifles [1,7,19,20]. As the loadsol^®^ in-shoe force sensors are calibrated using the wearer’s own body weight, the accuracy of the sensors may be affected by the additional load soldiers have to carry. It is also not known whether the structure and properties of the military boots would compromise the accuracy of the loadsol^®^ sensors.

Thus, the purpose of this study was to establish the validity of the loadsol^®^ in-shoe force sensors in military personnel during loaded walking on flat, inclined and declined surfaces. Specifically, the normal GRF variables recoded from loadsol^®^ during loaded walking (carrying 25 kg and 35 kg) would be compared with data obtained from an instrumented treadmill as the gold standard. Since military personnel tend to encounter more extreme terrains, such as walking up and down steep slopes, we used a higher gradient (10°, or 17.6%) than previous work (5.7°, or 10%) [18] to better reflect their occupational demands.

## 2. Materials and Methods

### 2.1. Study Design

This study conducted experiments on human participants in a laboratory. The test protocol involved walking on an instrumented treadmill on flat, inclined and declined gradients while carrying heavy loads. Normal GRF, perpendicular to the contact surface, was concurrently measured using two pieces of equipment (loadsol^®^ in-shoe force sensors and instrumented treadmill) for comparison.

### 2.2. Participants

Ethical approval was obtained from the Nanyang Technological University Institutional Review Board (Reference Number IRB-2022-0186). As this study focused on military application, we targeted full-time regular Singapore Armed Forces (SAF) personnel who were fit for duty. Specifically, the inclusion criteria were that participants were (1) men, (2) aged 21 to 45 years old, (3) full-time SAF personnel, and (4) currently healthy to perform route marches. Participants were excluded if they had any history of back or lower limb surgery. A total of 8 individuals who met all inclusion criteria were recruited, and all gave written informed consent to participate in the study. The physical characteristics of the participants are shown in Table 1.

### 2.3. Equipment

**Figure 1:**
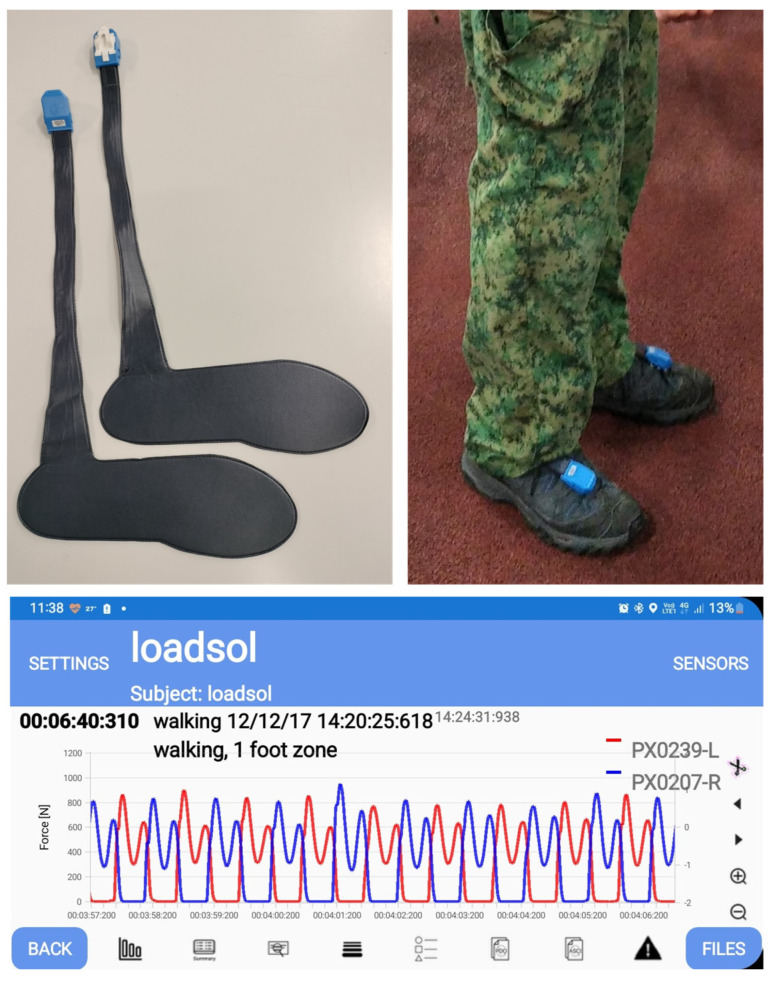
The loadsol^®^ wireless in-shoe sensors were fitted into participants’ military boots to measure normal ground reaction forces during walking via the loadsol-s mobile phone app (version 1.7.51).

The wireless loadsol^®^ insoles (novel, Munich, Germany) were used to measure the normal GRF under each foot of the participants during walking (see Figure 1). The insole sizes were matched to the participants’ feet sizes measured using a Brannock device. Normal GRF data were recorded via the loadsol-s version 1.7.51 at a 100 Hz scanning rate with the iPhone 11 (Apple, Cupertino, CA, USA). This app was used for both data acquisition and exporting of force-time data in ASCII format for further processing. While a newer version of loadsol^®^ allows a higher sampling rate of 200 Hz [18], our laboratory only possessed the earlier model with a maximum rate of 100 Hz. For walking activities in military boots, a sampling rate of 100 Hz has been used in other studies using in-shoe force [7] or pressure sensors [11]. Calibration of the sensors was performed using participants’ own body weight following the manufacturer’s guidelines. In brief, participants were asked to lift one foot such that their entire body weight was loaded onto the standing leg. A researcher did zeroing of the insole force on the app while the participant’s foot was off the ground and fully unloaded. The same calibration procedures were then repeated for the other foot.

The Bertec instrumented treadmill with two embedded force plates (Bertec Inc., Columbus, OH, USA) was used as a gold standard to validate the loadsol^®^ sensors. GRF in all three directions (normal, antero-posterior, medio-lateral) was recorded during walking trials on the treadmill. Only the normal GRF data were used in this validation study because loadsol^®^ sensors could only capture forces in this direction. The sampling rate of the treadmill GRF measurements was 1000 Hz, which was in alignment with other validation studies on loadsol^®^ using either force platforms or an instrumented treadmill as references [15,16,18].

### 2.4. Procedures

**Figure 2:**
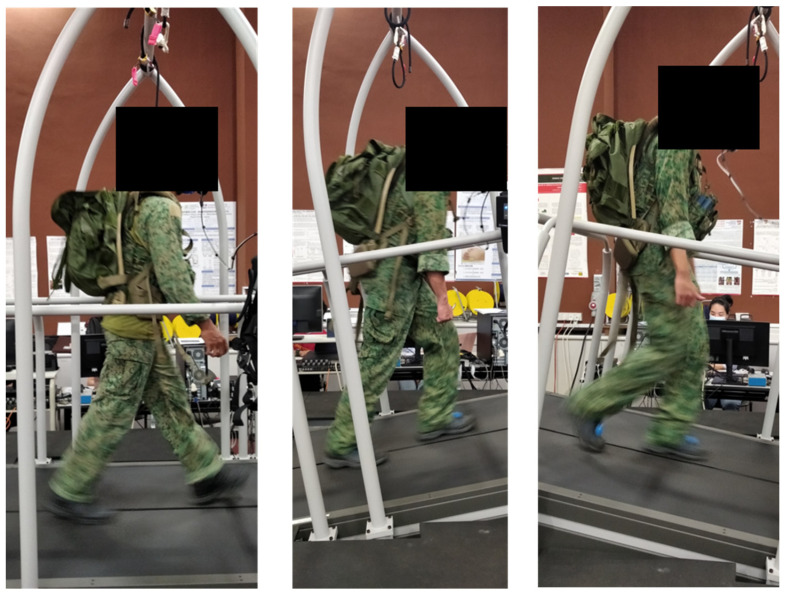
Participants carried heavy loads and walked on an instrumented treadmill on flat, 10° inclined and 10°declined 10° surfaces.

Participants walked on the instrumented treadmill in their military gear under two loads and three gradients (Figure 2). The loads (25 kg and 35 kg) and gradient (10°, or 17.6%) were selected to reflect military operational demands. The use of fixed loads instead of body weight percentages is common in military research because most equipment weights are standard and cannot be adjusted to the soldiers’ physical characteristics. The 25 kg and 35 kg loads chosen in the present study closely reflect the loads typically used in SAF training and operations in Singapore. These loads are comparable to other military studies in different countries; for example, Chatterjee et al. [21] used fixed loads of 10.7 kg, 21.4 kg, and 30 kg to examine Indian Army soldiers’ performance in high mountain terrain, while Lange et al. [20] used a standard 20 kg backpack and an assault rifle for all Swiss Army recruits. The walking speeds were set as 4 km/hr for flat, 2 km/h for inclined, and 3 km/hr for declined surfaces. We note that a previous study on loadsol^®^ validation used a fixed speed (1.3 m/s, or 4.68 km/h) for flat, inclined and declined surfaces, but this earlier study investigated only unloaded walking and used a gentler slope (5.7°, 10%). Considering the more extreme context in the military population, it would be unrealistic to set the same walking speed for flat and sloped walking while carrying heavy loads. Based on our experience and pilot work, we decided to use slower speeds for walking uphill (2 km/h) and downhill (3 km/h) compared with the flat surface (4 km/h). This approach offers good ecological validity while ensuring the safety of the participants. The test orders of the 6 different conditions (2 loads × 3 surfaces) were randomly assigned to the participants.

At the beginning of the test session, participants walked unloaded on the treadmill at 4 km/h for 5 min as a warm-up. A pair of size-matched loadsol^®^ sensors were then placed inside the military boots to measure in-shoe GRF forces under each foot. After standard calibration procedures, participants would then complete the 6 walking conditions in the assigned orders, lasting 10 min per condition. At the 9th min mark of each walking bout, in-shoe loadsol^®^ and treadmill GRF were concurrently collected for 30 s. This was achieved by manually triggering both systems to start recording at approximately the same time. While the two systems were not completely synchronised during data collection, precise matching of the data was done offline based on the heel strike and toe-off events detected from GRF measurements. Since participants were carrying relatively heavy loads of up to 35 kg, a long rest time of 10–15 min was given between trials to minimise fatigue and exhaustion. The entire data collection took approximately 3 h for each participant.

### 2.5. Data Processing

Normal GRF, perpendicular to the contact surface direction, were exported in ASCII format from both loadsol^®^ and the Bertec treadmill. Customised MATLAB codes were written for processing the raw GRF data (version 2022a, The MathWorks, Inc., Natick, MA, USA). For raw loadsol^®^ GRF data, no filtering procedures were applied due to the relatively low sampling frequency at 100 Hz [18]. Raw treadmill GRF signals were low-pass filtered using a 4th order Butterworth filter with a cut-off of 100 Hz to attenuate background higher frequency noise. This cut-off frequency was determined from residual analysis, and the value was in line with previous studies using the same treadmill model for gait analysis [22,23]. For each step, stance phase duration was determined as the time between touchdown and toe-off, with both events set at the detection threshold of 40 N. Data of crossover steps (i.e., when the foot was not wholly on its treadmill belt, with a part landing on the adjacent belt), indicated by a substantially reduced or prolonged stance phase duration, were also removed. Verification of the absence of crossover steps was further confirmed through visual inspection. Normal GRF data were also normalised to each participant’s body weight (BW) to facilitate comparison across individuals. GRF time graphs were generated based on a stance phase which was time-normalised (0–100%). Ensemble mean and standard deviation (SD) time series graphs of the normal GRF were calculated from the 8 participants for each walking condition.

From both sets of GRF data, impact and active peak forces were identified for each step. Loading rate, defined as the speed at which forces impact the body, was calculated using the 20–80% region of the phase from touchdown to impact peak force. Other variables extracted for both lower limbs included stance time (duration from a foot first touching the ground to the same foot leaving the ground), stride time (duration between the first contact of a foot and the following contact of the same foot), and impulse of the stance phase (area under the force-time graph). For each participant, the mean data of all usable steps within the 30 s data collection duration were used to represent a particular load and gradient condition. Cadence was determined by counting the number of steps (both left and right foot contacts) within the 30 s window. Step length was then calculated by dividing the walking speed by step frequency (derived from cadence), representing the average distance between the point of initial contact of one foot and the point of initial contact of the contralateral foot.

### 2.6. Statistical Analysis

A total of 8 variables of interest were compared between loadsol^®^ and the Bertec treadmill, including 4 kinetic (impact peak force, active peak force, impulse, and loading rate) and 4 spatiotemporal (stance time, stride time, cadence, step length) variables. Data from the left and right feet were analysed separately as there can be inherent bilateral differences in gait [24]. To assess the validity of the loadsol^®^ sensors, Bland–Altman plots [25] and 95% Limits of Agreement (LoA) were calculated for each variable under different loads (25 kg, 35 kg) and gradients (flat, inclined, declined). We plotted the differences (loadsol^®^-Bertec) against the reference method (Bertec) in the x-axis instead of taking the average of both methods as the x-axis [26]. Bias was calculated as the mean difference between the values obtained from loadsol^®^ and the instrumented treadmill. This statistical analysis approach has been widely used in other validation studies, including those specifically on the loadsol^®^ system [16,17,18]. Data are expressed as mean (standard deviation, SD) unless otherwise stated.

## 3. Results

**Figure 3:**
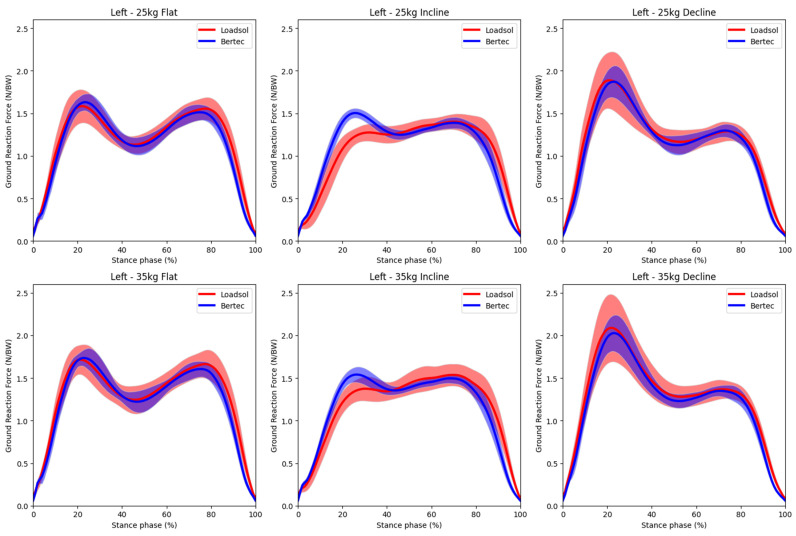
Comparison of the ensemble mean (solid line) and standard deviation (shaded region) of normal ground reaction forces (left foot contacts) concurrently measured using the loadsol^®^ in-shoe sensors (red) and the Bertec (blue) instrumented treadmill during walking on different gradients (flat, inclined, decline) while carrying different loads (25 kg, 35 kg). Data are time normalised to the stance phase (0–100%).

**Figure 4:**
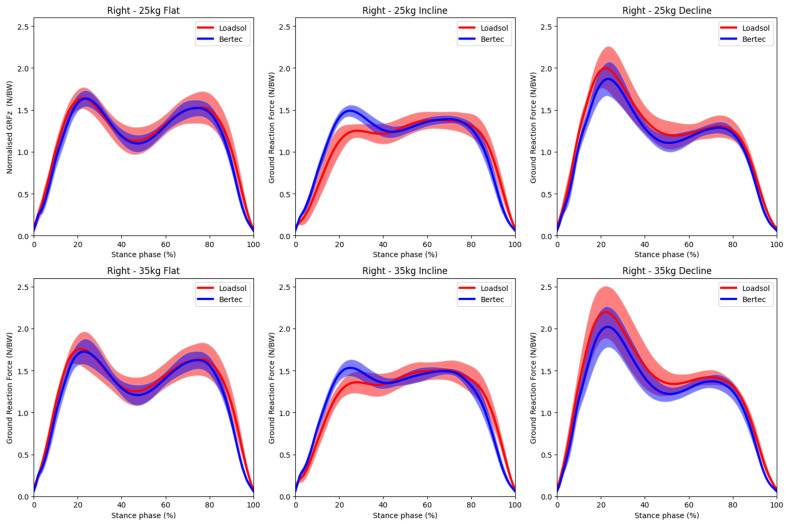
Comparison of the ensemble mean (solid line) and standard deviation (shaded region) of normal ground reaction forces (right foot contacts) concurrently measured using the loadsol^®^ in-shoe sensors (red) and the Bertec (blue) instrumented treadmill during walking on different gradients (flat, inclined, decline) while carrying different loads (25 kg, 35 kg). Data are time normalised to the stance phase (0–100%).

Visual comparisons of the normal GRF time graphs are illustrated in Figure 3 (left steps) and Figure 4 (right steps). The general force-time profiles were similar between the two pieces of equipment, with impact and active peaks clearly captured. However, the impact peak force measured using loadsol^®^ seemed consistently lower than the Bertec treadmill reference during the inclined walking conditions with both 25 kg and 35 loads. The spread of the ensembled mean (SD) graphs was quite large, indicating considerable inter-individual differences in the GRF patterns among the 8 participants.

**Figure 5:**
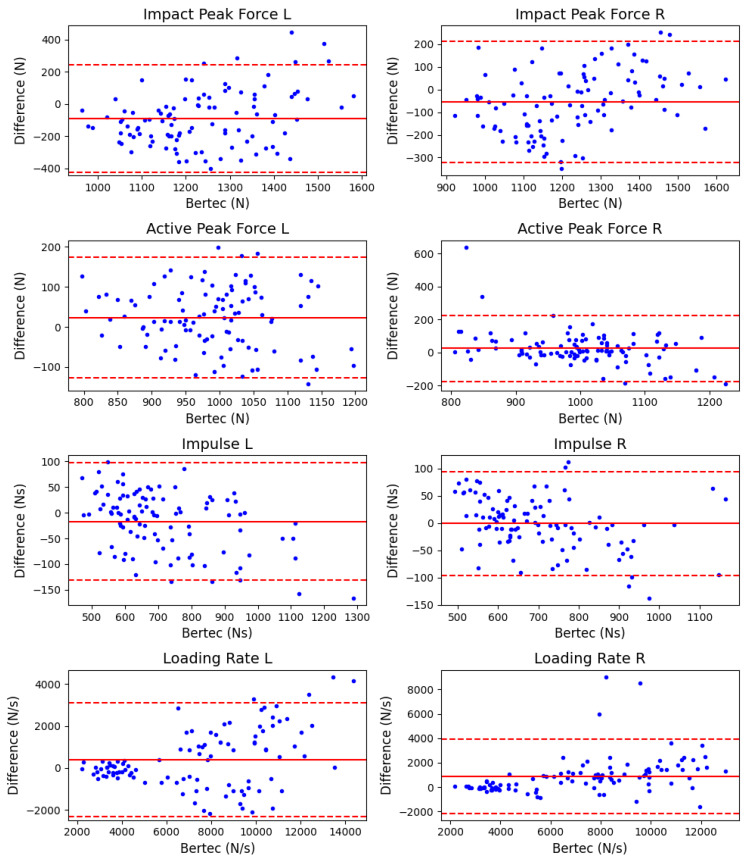
Bland-Altman plots of kinetic variables of the left (L) and right (R) foot contacts across all load and gradient conditions. The solid red line indicates the bias between the equipment (Difference = loadsol^®^ variables—Bertec treadmill variables), while the dashed red line indicates the 95% Limits of Agreement.

**Figure 6:**
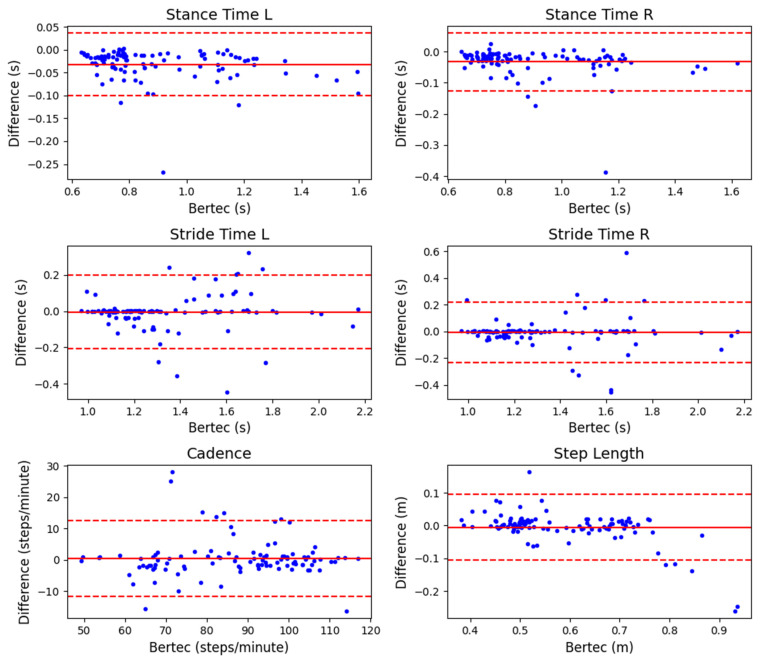
Bland-Altman plots of spatio-temporal variables of the left (L) and right (R) foot contacts across all load and gradient conditions. The solid red line indicates the bias between the equipment (Difference = loadsol^®^ variables—Bertec treadmill variables), while the dashed red line indicates the 95% Limits of Agreement.

The Bland-Altman plots for kinetic and spatiotemporal variables across all load and gradient conditions are shown in Figure 5 and Figure 6, respectively. The corresponding mean (SD) values, bias and 95% LoA are tabulated in Table 2 (flat surface), Table 3 (inclined surface), and Table 4 (declined surface). The bias of most kinetic and spatiotemporal variables was generally low, with a narrow range of LoA. These results indicate high accuracy and good agreement of the loadsol^®^ system with standard laboratory equipment. One interesting observation was that during inclined walking, loadsol^®^ tended to underestimate the impact peak force, but not during flat or declined walking. This trend was consistent for both 25 kg (Figure 3) and 35 kg (Figure 4) conditions. The mean bias of the impact peak force during inclined walking (183.4 to 188.6 N) was much higher than those under flat (−25.9 to −45.8 N) and declined (29.2 to 70.3 N) conditions (Table 2, Table 3 and Table 4).

## 4. Discussion

This study examined the validity of the loadsol^®^ in-shoe force sensors during loaded walking on flat, inclined and declined surfaces while wearing military boots and carrying heavy loads. Overall, the force-time profiles were similar between loadsol^®^ sensors and the instrumented treadmill, which serves as the gold standard. The generally low bias and good agreement with standard laboratory equipment for most kinetic and spatiotemporal variables indicate the high accuracy of the loadsol^®^ system. These results support that the loadsol^®^ force sensor insole is a valid tool for measuring normal GRF during walking in military boots under heavy load carriage on different gradients.

### 4.1. Kinetic Variables

We plotted the mean ensembled GRF time graph over the entire stance phase for all participants to allow a visual comparison of the force profile for each walking condition (Figure 3 and Figure 4). These graphs indicated that the loadsol^®^ sensors are able to clearly capture the two peak forces (impact peak and active peak) during walking even when sampled at 100 Hz, which was much lower than the Bertec treadmill’s rate of 1000 Hz. The large inter-individual differences in the GRF among the participants, as reflected by the SD of the graphs, could be due to variations in the magnitude of the forces or the timing at which the peak forces occurred. In the literature, no GRF time graphs comparing loadsol^®^ and any reference methods during walking were available for direct comparison. Burns and colleagues [15] used an instrumented treadmill to validate loadsol^®^ sensors during hopping, walking and running, but no GRF time graphs were shown. Others only plotted a single step of representative running strike to compare loadsol^®^ and force platforms embedded in the ground [16,18]. For running strikes, the impact peak force cannot be captured accurately by loadsol^®^ due to the low sampling rate; hence, previous work only analysed the active peak force [16]. To the authors’ best knowledge, this is the first study to illustrate the mean ensemble GRF time graphs obtained from loadsol^®^ sensors in comparison with laboratory standard equipment. Findings from the present study indicated that for walking at 2 to 4 km/h, the loadsol^®^ sensors sampling at 100 Hz is sufficient to allow analyses of both impact and active peak forces.

Four kinetic variables of interest were extracted for analysis in this study, namely impact peak force, active peak force, impulse and loading rate. These variables were commonly used in other studies on gait kinetics, military boots, and validation of loadsol^®^ sensors [10,11,15,16,17,18]. We observe relatively low bias and good agreement for most variables, paralleling the results from other studies comparing loadsol^®^ outputs with force platforms or instrumented treadmills [15,16]. For example, Sieberl et al. [16] reported a mean bias of 48 (83.4) N for active peak force during running. Similarly, Burns et al. [15] calculated the mean bias for peak GRF as 51.4 [50.2, 52.7] N for left foot steps and 28.7 [27.6, 30.1] N for right foot steps during walking. Active peak force data from the current study correspond well with the mean absolute bias of 2.0 to 51.6 N across all loading and walking surface conditions (Table 2, Table 3 and Table 4). For impulse measurement, Sieberl et al. [16] reported a mean bias of 5.6 (15.2) Ns for impulse measurements during running. Burns et al. [15] likely reported their impulse results during walking in the wrong unit of Nm. Assuming the authors were meant to use the unit Ns, the bias for impulse was 27.7 [27.3, 28.1] Ns and 20.0 [19.5, 20.4] Ns for the left and right steps, respectively. The results from the present study are in good agreement with the previous work, with mean bias ranging from 0.7 to 43.0 Ns for impulse across all tested conditions. Direct comparison of loading rates with the literature is difficult as previous work either did not report them [15] or used different methods to determine loading rates [16,17,18]. Taking these findings together, the loadsol^®^ sensors seem to offer a similar degree of accuracy when placed inside military boots compared with other types of footwear [15,17,18]. Neither was the validity of kinetic measurements compromised when participants carried heavy loads of 25 kg and 35 kg.

At a rather steep gradient of 10° (or 17.6%), loadsol^®^ tended to underestimate the impact peak force during inclined walking but not during flat or declined walking. This trend was consistent for both load conditions, with a higher mean bias of the impact peak force during inclined walking than flat and declined walking (Table 2, Table 3 and Table 4). One possible explanation is the foot movement inside the boot during dynamic tasks. During the process of loadsol^®^ calibration, the foot was placed in a normal position inside the boot, where most (if not all) contact forces were recorded by the sensors. During inclined walking, the foot position inside the boot may have shifted posteriorly, resulting in part of the heel falling outside of the loadsol^®^ insole boundary. Forces that acted outside the loadsol® insole cannot be registered, leading to a lower reading than the normal GRF. In the literature, only one other study compared the accuracy of loadsol^®^ when walking on different gradients [18]. The authors reported a significant bias in normal GRF between loadsol^®^ and the instrumented treadmill during inclined walking (bias = instrumented treadmill—loadsol^®^ = −0.73 BW) but not during flat (bias 0.02 BW) and declined (bias 0.03 BW) walking. It should be noted that the earlier study by Renner et al. [18] standardised the walking speed at 1.3 m/s across all gradients, while the present study used a much slower speed (2 km/h, or 0.55 m/s) of inclined walking under heavy load carriage. Regardless of the walking speed, both studies found that normal GRF during the early stance phase (e.g., near impact peak force) was less accurate when walking up an inclined slope as compared with flat and declined surfaces.

In the military context, one previous study used the loadsol^®^ sensors to compare in-shoe forces between different types of combat boots [7]. This earlier study included a loaded walking condition where participants carried a 20 kg field pack but did not check the validity of the sensors under heavy load situations. The present study provided empirical data to show good accuracy of the loadsol^®^ force sensors during loaded walking. This reassures that inserting the loadsol® force insoles into combat boots is appropriate to measure in-shoe forces during loaded walking, as performed in the previous study [7]. Having established the validity of this commercially available system also creates opportunities for other military applications, such as monitoring the gait characteristics during a route march. The current loadsol^®^ system requires a mobile device, such as a mobile phone or an iPad, to be placed in close proximity for data acquisition. Future innovation may explore ways to wirelessly transfer real-time data via cloud terminal and to comprehensively extract meaningful parameters for health and performance monitoring [27].

### 4.2. Spatiotemporal Variables

We analysed four spatiotemporal variables directly extracted or calculated from GRF data: stance time, stride time, cadence, and step length. Similar to the kinetic variables, the bias was generally low, and the LoA range was small. These results indicate good accuracy of the loadsol^®^ sensors in measuring gait variables associated with time. In a previous study comparing loadsol^®^ with an instrumented treadmill, no spatiotemporal variables were investigated [18]. Using the force platform embedded in the ground, one study reported a mean bias of 0.00 s (both left and right feet) in ground contact time during walking at 5 km/h [15]. Another study on running found that the loadsol^®^ slightly overestimated ground contact time with a small mean bias of 2.3 ms (or 0.0023 s) [16]. Parallel to these previously reported results, our data also showed very small bias ranging from 0.009 s to 0.038 s in stance time (same as ground contact time) across different load and gradient conditions, with most bias falling below 0.02 s. There are no reported validity data on stride time, cadence, or step length in the literature for direct comparison. The lack of previous validity data on key spatiotemporal variables highlighted the novelty of the present study to comprehensively evaluate the loadsol^®^ sensors.

### 4.3. Limitations

Some limitations of this study should be considered. First, our sample size of 8 participants is quite small because we targeted specialised military applications. For good ecological validity, participants had to be full-time army personnel familiar with military equipment and physically fit to walk for a prolonged period while carrying heavy loads of up to 35 kg. These strict criteria and test protocols made the study demanding and limited us to fewer participants. Second, we did not standardise the military boots used because each participant has their own preferred boot models. Given the high physical demand of the protocol, it is important that participants walk in a comfortable pair of boots that they are used to. While some differences in GRF may be expected between walking in different combat boots [7], the small variations in boots should not have affected the validity results as the bias observed in the present study was comparable to other studies using running shoes [17,18] or tight-fitted socks without shoes [16].

## 5. Conclusions

This study established the validify of loadsol^®^ in-shoe force sensors for normal ground reaction force measurement in military personnel with consideration of their occupational demands, such as the need to carry heavy loads, wear protective, military boots, and traverse steep slopes. When compared with the instrumented treadmill sampling at 1000 Hz, the loadsol^®^ sensors were able to record similar force-time profiles over the entire stance phase and clearly capture both impact and active peak forces during walking at a lower sampling rate of 100 Hz. In general, low bias and good agreement were observed for most kinetic and spatiotemporal variables as compared with standard laboratory equipment. These validation results demonstrate that the loadsol^®^ sensors, when inserted into military boots, are accurate for GRF measurements under demanding situations such as traversing steep slopes while carrying heavy loads. Considering the portability and ease of use of loadsol^®^ in-shoe sensors, future work can apply these sensors for in-field measurements during military operations and related tasks.

## Figures and Tables

**Table 1:** Physical characteristics of 8 male full-time military personnel.

	Mean (SD)	Range
Age (years)	32.4 (5.8)	25–40
Body Mass (kg)	69.8 (4.4)	61.4–74.7
Height (cm)	170.0 (5.5)	162.6–178.5
Shoe Size (European, EU)	41.5 (1.5)	40–45

**Table 2:** Comparison of loadsol^®^ and Bertec instrumented treadmill during walking on a flat surface while carrying heavy loads.

Variables	25 kg Load			35 kg Load		
	loadsol^®^	Bertec	Bias (95% LoA)	loadsol^®^	Bertec	Bias (95% LoA)
Stance Time L (s)	0.76 (0.07)	0.77 (0.07)	−0.009 (−0.02–0.01)	0.76 (0.04)	0.77 (0.04)	−0.009 (−0.02–0.0)
Stance Time R (s)	0.75 (0.06)	0.76 (0.06)	−0.011 (−0.02–0.0)	0.76 (0.04)	0.78 (0.04)	−0.016 (−0.07–0.04)
Stride Time L (s)	1.18 (0.1)	1.18 (0.1)	0.001 (−0.01–0.01)	1.18 (0.06)	1.2 (0.05)	−0.027 (−0.1–0.05)
Stride Time R (s)	1.18 (0.1)	1.19 (0.1)	−0.004 (−0.02–0.01)	1.18 (0.07)	1.23 (0.1)	−0.048 (−0.25–0.16)
Cadence (steps/minute)	96.0 (7.6)	96.3 (8.7)	−0.3 (−3.8–3.3)	94.7 (5.1)	93.28 (4.93)	1.423 (−7.23–10.08)
Step Length (m)	0.70 (0.06)	0.70 (0.07)	0.00(−0.03–0.03)	0.71 (0.04)	0.72 (0.04)	−0.01 (−0.08–0.06)
Impact Peak Force L (N)	1108.0 (136.0)	1153.8 (79.8)	−45.8 (−280.1–188.6)	1204.9 (113.8)	1233.7 (70.7)	−28.7 (−243.4–185.9)
Impact Peak Force R (N)	1125.1 (88.3)	1151.0 (86.3)	−25.9 (−192.9–141.1)	1217.1 (82.81)	1218.2 (78.5)	−1.1 (−132.8–130.6)
Active Peak Force L (N)	1074.1 (33.6)	1022.6 (52.0)	51.6 (−63.5–166.6)	1141.4 (58.0)	1072.2 (59.1)	69.1 (−88.3–226.6)
Active Peak Force R (N)	1050.4 (52.5)	1032.7 (54.3)	17.6 (−155.3–190.6)	1123.6 (60.1)	1086.7 (72.5)	37.0 (−146.7–220.6)
Impulse L (Ns)	617.8 (68.6)	599.4 (73.6)	18.4 (−20.6–57.5)	665.5 (42.2)	648.9 (51.3)	16.7 (−52.9–86.2)
Impulse R (Ns)	615.8 (78.2)	593.5 (59.0)	22.3 (−50.3–94.9)	670.3 (51.4)	651.0 (43.1)	19.3 (−45.1–83.7)
Loading Rate L (N/s)	8150.2 (2562.6)	7602.2 (1697.9)	548.0 (−1472.9–2569.0)	9080.1 (2583.3)	8522.5 (1359.9)	557.6 (−1980.3–3095.6)
Loading Rate R (N/s)	7895.1 (1088.3)	7092.9 (1117.7)	802.1 (288.6–1315.7)	9207.8 (1196.1)	8264.4 (1410.0)	943.5 (345.5–1541.5)

Note. L denotes left, R denotes right, and LoA denotes limits of agreement. Bias was calculated as the mean difference between the values obtained from loadsol^®^ and instrumented treadmill.

**Table 3:** Comparison of loadsol^®^ and Bertec instrumented treadmill during walking on a 10° inclined surface while carrying heavy loads.

Variables	25 kg Load			35 kg Load		
	loadsol^®^	Bertec	Bias (95% LoA)	loadsol^®^	Bertec	Bias (95% LoA)
Stance Time L (s)	1.18 (0.24)	1.22 (0.26)	−0.034 (−0.09–0.03)	1.13 (0.14)	1.15 (0.16)	−0.026 (−0.08–0.02)
Stance Time R (s)	1.18 (0.24)	1.2 (0.25)	−0.027 (−0.05–−0.0)	1.14 (0.15)	1.15 (0.16)	−0.015 (−0.05–0.02)
Stride Time L (s)	1.71 (0.3)	1.71 (0.32)	−0.003 (−0.08–0.07)	1.69 (0.25)	1.64 (0.2)	0.05 (−0.21–0.31)
Stride Time R (s)	1.72 (0.3)	1.71 (0.32)	0.013 (−0.09–0.12)	1.73 (0.3)	1.63 (0.21)	0.102 (−0.33–0.54)
Cadence (steps/minute)	63.9 (12.0)	66.2 (13.6)	−2.31 (−7.71–3.1)	65.8 (11.46)	68.7 (9.5)	−2.9 (−13.5–7.7)
Step Length (m)	0.54 (0.1)	0.53 (0.11)	0.02 (−0.02–0.06)	0.52 (0.09)	0.5 (0.07)	0.03 (−0.08–0.14)
Impact Peak Force L (N)	885.5 (42.6)	1068.8 (68.7)	−183.4 (−328.4–−38.4)	951.3 (65.8)	1089.1 (73.0)	−137.8 (−268.5–−7.0)
Impact Peak Force R (N)	872.8 (45.2)	1061.4 (69.8)	−188.6 (−336.5–−40.7)	950.0 (61.8)	1084.0 (89.0)	−134.0 (−290.0–21.9)
Active Peak Force L (N)	957.2 (27.5)	953.0 (55.9)	4.2 (−121.5–129.9)	1055.2 (47.5)	1011.9 (60.9)	43.3 (−134.3–220.9)
Active Peak Force R (N)	952.0 (44.1)	954.9 (49.2)	−2.9 (−143.2–137.4)	1034.9 (55.2)	1020.7 (57.6)	14.2 (−151.2–179.6)
Impulse L (Ns)	857.1 (165.8)	856.4 (178.1)	0.7 (−71.5–72.8)	891.4 (124.6)	882.8 (128.7)	8.6 (−97.0–114.2)
Impulse R (Ns)	850.5 (192.8)	847.8 (172.2)	2.8 (−102.1–107.6)	896.2 (152.0)	885.8 (138.0)	10.4 (−95.8–116.6)
Loading Rate L (N/s)	3342.2 (884.9)	3420.4 (1123.7)	−78.2 (−699.2–542.9)	3750.2 (549.4)	3796.5 (652.5)	−46.3 (−659.9–567.3)
Loading Rate R (N/s)	3729.1 (1001.9)	3690.0 (1024.1)	39.1 (−1001.3–1079.5)	3909.7 (642.2)	4056.2 (786.1)	−146.5 (−767.2–474.2)

Note. L denotes left, R denotes right, and LoA denotes limits of agreement. Bias was calculated as the mean difference between the values obtained from loadsol^®^ and instrumented treadmill.

**Table 4:** Comparison of loadsol^®^ and Bertec instrumented treadmill during walking on a 10° declined surface while carrying heavy loads.

Variables	25 kg Load			35 kg Load		
	loadsol^®^	Bertec	Bias (95% LoA)	loadsol^®^	Bertec	Bias (95% LoA)
Stance Time L (s)	0.74 (0.14)	0.78 (0.14)	−0.038 (−0.09–0.02)	0.71 (0.09)	0.74 (0.09)	−0.027 (−0.1–0.04)
Stance Time R (s)	0.73 (0.12)	0.75 (0.11)	−0.015 (−0.04–0.01)	0.72 (0.09)	0.74 (0.11)	−0.021 (−0.07–0.03)
Stride Time L (s)	1.16 (0.18)	1.19 (0.18)	−0.029 (−0.13–0.08)	1.13 (0.13)	1.16 (0.15)	−0.023 (−0.24–0.2)
Stride Time R (s)	1.17 (0.19)	1.18 (0.19)	−0.017 (−0.08–0.05)	1.15 (0.13)	1.12 (0.14)	0.029 (−0.13–0.19)
Cadence (steps/minute)	97.9 (14.0)	96.2 (15.1)	1.7 (−4.8–8.2)	99.7 (12.12)	100.0 (12.7)	−0.2 (−15.9–15.4)
Step Length (m)	0.54 (0.09)	0.55 (0.1)	−0.01 (−0.05–0.03)	0.52 (0.07)	0.52 (0.07)	0.00 (−0.07–0.07)
Impact Peak Force L (N)	1329.3 (203.7)	1300.1 (92.8)	29.2 (−321.5–380.0)	1438.2 (231.1)	1394.4 (87.7)	43.8 (−356.2–443.8)
Impact Peak Force R (N)	1366.0 (117.6)	1295.7 (105.3)	70.3 (−96.8–237.4)	1532.4 (121.1)	1406.7 (109.2)	125.8 (−22.0–273.5)
Active Peak Force L (N)	888.2 (57.2)	890.2 (50.87)	−2.0 (−99.2–95.2)	938.7 (68.9)	926.1 (67.6)	12.6 (−70.3–95.5)
Active Peak Force R (N)	893.0 (42.6)	879.0 (54.8)	14.1 (−110.5–138.7)	1055.6 (173.6)	935.9 (86.1)	119.7 (−304.4–543.8)
Impulse L (Ns)	601.3 (126.5)	587.3 (119.9)	14.0 (−70.5–98.5)	623.0 (86.6)	608.0 (87.0)	15.0 (−81.1–111.2)
Impulse R (Ns)	609.0 (115.1)	566.1 (89.0)	43.0 (−41.6–127.5)	662.6 (89.0)	617.7 (95.0)	44.9 (2.3–87.6)
Loading Rate L (N/s)	10,857.3 (2657.7)	10,183.7 (1568.7)	673.6 (−2785.6–4132.7)	11,515.0 (3249.9)	10,523.6 (2320.6)	991.4 (−3286.3–5269.1)
Loading Rate R (N/s)	10,607.1 (1635.8)	9082.5 (1687.7)	1524.6 (222.7–2826.5)	11,989.4 (2344.8)	10,156.6 (2073.7)	1832.8 (340.4–3325.2)

Note. L denotes left, R denotes right, and LoA denotes limits of agreement. Bias was calculated as the mean difference between the values obtained from loadsol^®^ and instrumented treadmill.

## Data Availability

Data are available at the NIE Data Repository [https://doi.org/10.25340/R4/FUBH6F0].
